# Fukushima study for Engaging people with type 2 Diabetes in Behaviour Associated Change (FEEDBACK): study protocol for a cluster randomised controlled trial

**DOI:** 10.1186/s13063-023-07345-6

**Published:** 2023-05-08

**Authors:** Thomas Rouyard, Mei Endo, Ryota Nakamura, Michiko Moriyama, Maham Stanyon, Satoshi Kanke, Koki Nakamura, Cynthia Chen, Yasushi Hara, Masako Ii, Ryuki Kassai

**Affiliations:** 1 Research Center for Health Policy and Economics, Hitotsubashi University, 2-1 Naka, Kunitachi, Tokyo 186-8601 Japan; 2 Department of Community and Family Medicine, Fukushima Medical University, 1 Hikarigaoka, Fukushima, 960-1247 Japan; 3 Graduate School of Economics, Hitotsubashi University, 2-1 Naka, Kunitachi, Tokyo 186-8601 Japan; 4 Division of Nursing Science, Graduate School of Biomedical and Health Sciences, Hiroshima University, 1-2-3 Kasumi, Minami-ku, Hiroshima City, Hiroshima 734-8553 Japan; 5 Saw Swee Hock School of Public Health, National University of Singapore, 12 Science Drive 2, #10-01, Singapore, 117549 Singapore; 6 Graduate School of Business Administration, Kobe University, 2-1 Rokkōdaichō, Nada Ward, Kobe, Hyogo 657-0013 Japan

**Keywords:** Type 2 diabetes, Self-management, Behavioural intervention, Risk communication, Heart age, Doctor-patient relationship, Doctor-patient communication, Behavioural contract, Primary care, Cluster randomised trial

## Abstract

**Background:**

The growing burden of type 2 diabetes mellitus (T2DM) and the rising cost of healthcare worldwide make it imperative to identify interventions that can promote sustained self-management behaviour in T2DM populations while minimising costs for healthcare systems. The present FEEDBACK study (Fukushima study for Engaging people with type 2 Diabetes in Behaviour Associated Change) aims to evaluate the effects of a novel behaviour change intervention designed to be easily implemented and scaled across a wide range of primary care settings.

**Methods:**

A cluster randomised controlled trial (RCT) with a 6-month follow-up will be conducted to evaluate the effects of the FEEDBACK intervention. FEEDBACK is a personalised, multi-component intervention intended to be delivered by general practitioners during a routine diabetes consultation. It consists of five steps aimed at enhancing doctor-patient partnership to motivate self-management behaviour: (1) communication of cardiovascular risks using a ‘heart age’ tool, (2) goal setting, (3) action planning, (4) behavioural contracting, and (5) feedback on behaviour. We aim to recruit 264 adults with T2DM and suboptimal glycaemic control from 20 primary care practices in Japan (cluster units) that will be randomly assigned to either the intervention or control group. The primary outcome measure will be the change in HbA1c levels at 6-month follow-up. Secondary outcome measures include the change in cardiovascular risk score, the probability to achieve the recommended glycaemic target (HbA1c <7.0% [53mmol/mol]) at 6-month follow-up, and a range of behavioural and psychosocial variables. The planned primary analyses will be carried out at the individual level, according to the intention-to-treat principle. Between-group comparisons for the primary outcome will be analysed using mixed-effects models. This study protocol received ethical approval from the research ethics committee of Kashima Hospital, Fukushima, Japan (reference number: 2022002).

**Discussion:**

This article describes the design of a cluster RCT that will evaluate the effects of FEEDBACK, a personalised, multicomponent intervention aimed at enhancing doctor-patient partnership to engage adults with T2DM more effectively in self-management behaviour.

**Trial registration:**

The study protocol was prospectively registered in the UMIN Clinical Trials Registry (UMIN-CTR ID UMIN000049643 assigned on 29/11/2022). On submission of this manuscript, recruitment of participants is ongoing.

**Supplementary Information:**

The online version contains supplementary material available at 10.1186/s13063-023-07345-6.

## Background

It has been well established that sustained glycaemic control reduces the risk of microvascular complications [1] and is strongly associated with lower risks of cardiovascular (CV) complications and mortality in type 2 diabetes mellitus (T2DM) populations [2–5]. Estimations suggest that each absolute 1% (11 mmol/mol) reduction in haemoglobin A1c (HbA1c) decreases those risks by around 37% [5], 15–20% [5, 6], and 15% [5], respectively.

In line with these findings, achieving sustained control of HbA1c levels has become the cornerstone of T2DM management. While maintaining HbA1c below 7.0% (53 mmol/mol) is considered a reasonable goal for most adults with T2DM, international guidelines recommend adjusting target values on an individual basis [7, 8]. According to provider judgement and patient preference, lower values may be targeted if they can be achieved safely, while less stringent goals may be preferred to reduce the risk of treatment adverse events (e.g. in patients with severe co-morbidities).

In practice, however, many patients encounter difficulties in meeting their glycaemic target. In the US, it is estimated that 43% of insured people who were prescribed with oral hypoglycaemic agents in 2012 had suboptimal glycaemic control [9]; while, between 2006 and 2013, the proportion of privately- or Medicare-insured patients with HbA1c ≥9% increased from 9.9 to 12.2% [10]. Although several biological and psychosocial variables are known to influence HbA1c, evidence has shown that successful glycaemic control strongly depends on how closely patients follow diabetes self-management recommendations, which include regular physical exercise [11], healthy diet [12], self-monitoring of blood glucose (SMBG) [13], and adherence to medications [14]. In this paper, we follow Hood and colleagues (2015) and use the term ‘behavioural management’ to refer to such self-care behaviours [15]. In addition to contributing to lowering HbA1c, behavioural management also produces beneficial effects on other biomarkers associated with cardiovascular diseases (CVD), such as blood pressure and LDL cholesterol levels [14].

Over the past two decades, the search for strategies to improve behavioural management has attracted considerable attention [16, 17]. Numerous, multilevel factors have been identified as having an influence on the behaviour change process — including factors at the individual level (e.g. disease knowledge, health literacy, distress), environmental level (e.g. family support, relationship with provider), and institutional level (e.g. structural barriers to care access) [18] — which renders the design of effective interventions complex. This complexity is also reflected in the variety of health behaviour theories that have been used to underpin such interventions in the literature (for a review, see Peyrot and Rubin, 2007 [19]).

While purely educational support (sometimes referred to as ‘didactic education’, or ‘you should do’ approach) has only produced modest effects, if any, on behavioural or clinical outcomes [20], findings from health psychology and behavioural research have informed the development of more effective interventions. For example, it has been shown that people’s intentions to engage in behavioural management, which predict actual behaviour [21], are mediated by personal beliefs, such as perceived diabetes-related risks, perceived effectiveness of preventative strategies, and perceived personal control and self-efficacy [19]. Psychosocial interventions that have targeted such beliefs, such as motivational interviewing [22] or cognitive behavioural therapies [23], have produced greater effects on behaviour and glycaemic control, at least in the short term [24].

However, while adequate intentions are a prerequisite to behaviour change, they often are insufficient to achieve sustained behavioural management. Evidence suggests the existence of an *intention-behaviour gap*, whereby some patients fail to initiate and maintain behavioural management despite intending to [25]. Research in behavioural science has guided the development of interventions that embed multiple behaviour change strategies (or *behaviour change techniques* (BCT), according to Michie et al.’s taxonomy [26]) aimed at bridging this gap [27–29]. In the context of behavioural management, some BCTs appear to be more promising than others. For example, interventions that include ‘action planning’, ‘problem solving’, ‘feedback on behaviour’, ‘goal setting (behaviour)’, or ‘goal review’ components have been associated with greater change in diet and physical activity [27–29]. Furthermore, the BCTs ‘instruction on how to perform a behaviour’ and ‘action planning’ have been associated with significant improvements in glycaemic control (>0.3%) [27].

In addition to producing greater effects on behavioural outcomes, some of these multi-component interventions have also demonstrated long-term reductions in HbA1c [27, 30], which constitutes a major step forward in the management of T2DM. Nevertheless, concerns may be raised about how implementable and scalable such interventions are, as the most successful ones tend to be intense, multifaceted programmes involving an extensive team of caregivers and providing follow-up support over a long period of time [31–33]. For example, the long-term HbA1c reductions reported in the Look AHEAD trial resulted from an intense lifestyle intervention in which participants were seen at very frequent intervals over 4 years [32, 33]. Given the growing burden of T2DM [34] and the rise in healthcare costs worldwide [35], many healthcare systems are not equipped to support such programmes. Less costly but nonetheless effective interventions that build on available resources and existing infrastructure, i.e. interventions that can be more easily embedded into a wider range of clinical settings at scale, are urgently needed.

### Objectives

The primary objective of this cluster randomised controlled trial (RCT) is to evaluate the effects of a personalised, multicomponent intervention (‘FEEDBACK’) aimed at engaging adults with T2DM in behavioural management more effectively. FEEDBACK is a resource-minimising intervention designed to be delivered by general practitioners/family physicians (GPs) in primary care settings. Primary endpoints of the study will be the change in HbA1c levels at 6-month follow-up.

The secondary objective is to examine whether the FEEDBACK intervention increases the quality of the doctor-patient partnership, i.e. whether it improves doctor-patient communication and participatory decision-making from a patient perspective. Finally, the tertiary objective is to investigate the effects of FEEDBACK on a range of patients’ psychological outcomes.

## Methods

Study design, methods and results will be reported in accordance with the Consolidated Standards of Reporting Trials (CONSORT) guidelines extension for cluster trials [36].

### Design

The study is a superiority trial using a prospective, parallel-group, two-arm, cluster randomised controlled design with a 6-month follow-up, comparing the FEEDBACK intervention to a control intervention at the individual level. A cluster design with GP practice as the unit of randomisation is required for three reasons:

- GP practice randomisation will facilitate the implementation of the study by reducing the administrative burden for practice staff. Data management will be simplified (e.g. providers will only need to manipulate one type of questionnaire), resulting in a minimisation of the risk of measurement errors.
- Individual randomisation would render the providers’ task more difficult with the delivery of two different types of interventions. Practice randomisation will contribute to increase treatment delivery consistency and to reduce the risk of treatment allocation error.
- This will also allow potential contamination between study groups to be reduced. Assuming individual randomisation, there is a risk that participants allocated to different groups within the same practice may discuss and compare their respective interventions, resulting in an intra-correlation and possible requests to switch group.

Funding for this study was awarded by the Japan Society for the Promotion of Science (Grant Number 20K18934) and ethical approval was obtained from the research ethics committee of Kashima Hospital, Fukushima (reference number: 2022002). The study protocol has been registered in the UMIN Clinical Trials Registry (UMIN-CTR ID UMIN000049643 assigned on 29/11/2022 – https://center6.umin.ac.jp/cgi-open-bin/ctr_e/ctr_his_list.cgi?recptno=R000056537) and follows the recommendations of the SPIRIT guidelines [37]. A SPIRIT checklist with references to the relevant page numbers of this protocol is provided in supplementary materials.

### Setting

The study will take place in Fukushima and 14 other prefectures in Japan (Hokkaido, Aomori, Akita, Yamagata, Saitama, Kanagawa, Ishikawa, Osaka, Mie, Nara, Hyogo, Ehime, Oita, and Okinawa). Study setting will be GP practices. In Japan, as there is no clear distinction between primary and secondary care, patients with T2DM can visit any medical institution on a regular basis. In order to standardise the quality of care for this study, we will only recruit practices where GPs have been certified by the Japan Primary Care Association (JPCA), a member organisation of the World Organization of Family Doctors (WONCA).

### Recruitment and eligibility

#### General practitioners

GP partners will be invited to participate in the study by the research team, and those expressing an interest will be recruited. Eligible practices will need to have at least one JPCA-Certified Family Physician willing to participate in the study.

#### Individual participants

Potential participants will be identified through screening of practice records by the practice staff. GPs will review the generated lists to make sure that only individuals meeting the eligibility criteria are included (see Table 1). Reasons for exclusion will be recorded. The practice staff will contact potential participants by mail and/or telephone to introduce the research and to invite them to participate in the study.

**Table 1:** Inclusion and exclusion criteria

	**Inclusion criteria**	**Exclusion criteria**
GP practices	• At least one JPCA-Certified Family Physician willing to participate in the study	
Individual participants	• Adult aged between 20 and 69 years • Diagnosed as T2DM with HbA1c ≥ 7.0% at the last routine clinical check • Capable to fill in questionnaires in Japanese • Written informed consent	• Insulin users • Co-morbidities or disorders less compatible with behavioural management at the time of the study (e.g. cancer, substance abuse, significant mental health disorders) • Learning or communication difficulties

**Fig. 1:**
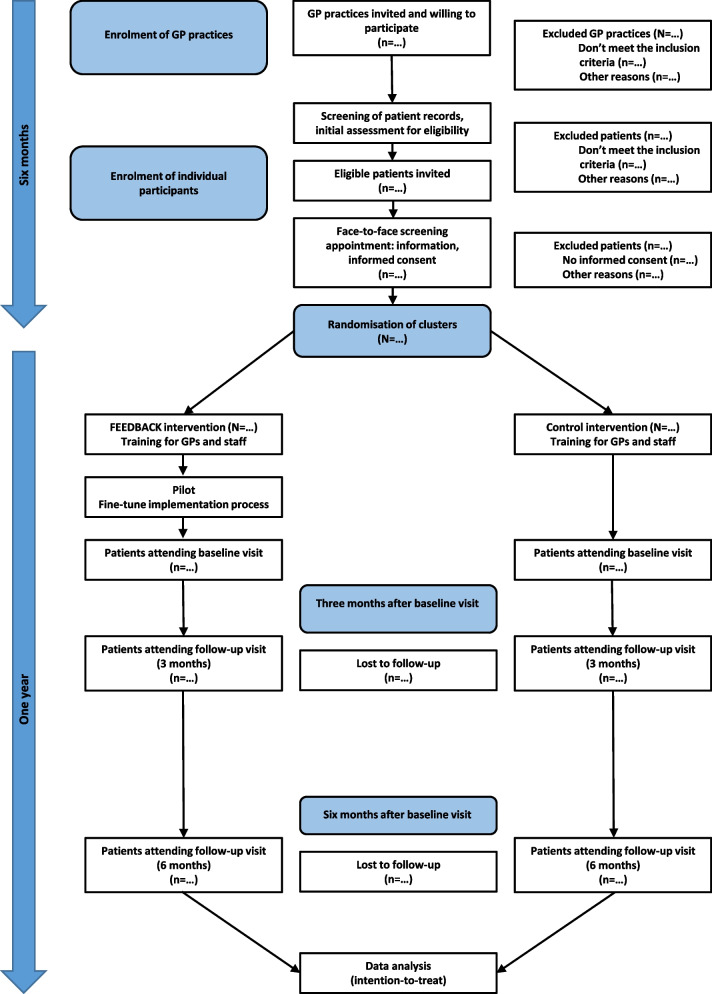
Study flow

Patients who express an interest in participating will be invited to attend a screening appointment at the practice, where the study will be fully explained and discussed with their GP. It will be clearly stated that the study is optional and that participants are free to withdraw at their own request and at any time, without prejudice to future care. Patients will also be informed that the allocation of their practice to intervention or control group will be random. Informed consent will be secured from all eligible participants willing to participate following this face-to-face introduction to the research (see study flow in Fig. 1).

### Randomisation

Randomisation of practices will be conducted by an independent member of the research team who will not be involved in data collection. Practices will be randomly allocated using a stratification method to minimise imbalances across intervention groups within each practice size (based on the number of actual study participants). Within each stratum, one practice will be randomly allocated to the intervention group and the other to the control group (allocation ratio of 1:1). The allocation sequence will be based on a computer-generated random number, using random permutated blocks with a block size of two.

Randomisation of practices will be conducted after completion of the recruitment of individual participants to reduce the risk of selection bias (sometimes also referred to as identification/recruitment bias in this context), in accordance with international guidelines for the conduct of cluster RCTs [38]. Indeed, if clusters were to be allocated to intervention or control prior to the recruitment of individual participants, there would be an increased risk of selection bias due to a priori knowledge of the allocation status. For example, a cluster allocated to intervention may be more likely to recruit participants who will tend to respond more positively to the intervention [39–41].

### Study interventions

#### Intervention group (FEEDBACK intervention)

The FEEDBACK intervention is a personalised, multi-component intervention developed based on Peyrot and Rubin’s ‘5C’ framework (see Table 2) [19]. This framework aims to guide the development of behaviour change programmes that can be delivered in routine diabetes care. It considers not only the efficacy of the programme, but also its practical aspects. The framework consists of a step-by-step approach, consistent with a number of health behaviour theories (see Peyrot and Rubin, 2007 [19]), in which five steps occur in a sequential order (5C):

1. *Constructing a problem definition*, i.e. identifying and specifying the patient’s problem
2. *Collaborative goal setting*, which involves concrete actions (e.g. ‘not snacking after dinner’) rather than values (e.g. ‘eating healthier’)
3. *Collaborative problem solving*, i.e. formulating strategies to overcome identified barriers and achieve the target goal(s)
4. *Contracting for change*, i.e. committing to the selected goals and strategies
5. *Continuing support*, i.e. assessing and updating the goals and strategies according to actual behaviour change

**Table 2:** Components of the FEEDBACK intervention

**Intervention components**	**Behaviour change techniques**^a^
***Step 1 – Constructing a problem definition***	
Assessing and communicating personalised CV risks using a ‘heart age’ tool	5.1. Information about health consequences 5.2. Salience of consequences
Identifying behavioural barriers and/or facilitators	1.2. Problem-solving
***Step 2 – Collaborative goal setting***	
Defining a realistic but challenging behaviour change goal	1.1. Goal setting (behaviour)
***Step 3 – Collaborative problem solving / action planning***	
Defining a realistic but challenging behaviour change strategy	1.2. Problem-solving 1.4. Action planning
***Step 4 – Contracting for change***	
Establishing and committing to a behavioural contract	1.8. Behavioural contract
***Step 5 – Continuing support***	
Communicating updated CV risks using the heart age tool	2.7. Feedback on outcome(s) of behaviour
Revising the behaviour change goal and strategy, if needed	1.5. Review behaviour goal(s)

^a^Coded according to Michie et al.’s Behavior Change Technique Taxonomy (v1) [26]

The 5C framework is rooted in the empowerment (or patient-centred) approach to care, which suggests that the patient should be at the centre of the behaviour change process [42]. As part of this approach, providers have a key role to play in fostering patient motivation and empowerment to adhere to behavioural management.

##### Risk communication

A central element of the FEEDBACK intervention is the use of an intuitive risk marker to increase patients’ perceived need to engage with behavioural management and to provide feedback on behaviour change [43]. In order to convey the risk of experiencing diabetes-related CV complications, which is often underestimated in T2DM populations [44], participants will be communicated their ‘effective heart age’ [45]. This risk metric corresponds to the age of a similar, ‘well-controlled’ individual (i.e. whose risk factors are within normal value ranges) with matching CV risk probability [46]. Such an intuitive risk format aims to make the risk message more salient and memorable as compared to probabilistic risk information, which tends to be poorly understood by patients [47, 48]. Participants’ effective heart age will be calculated using PERCODIA [49], a tool specifically developed for intuitive risk communication to people with T2DM in routine diabetes care.

##### Goal setting

Based on identified behavioural barriers and/or facilitators, participants and GPs will agree on a personalised behaviour change goal. The goal setting process must be SMART, i.e. the selected goal will need to be specific, measurable, achievable, realistic, and time-bound [50].

##### Action planning

According to the goal set, a specific action plan will also be mutually agreed upon. This action plan will consist of concrete, measurable behavioural changes that need to be implemented until the next visit (i.e. on average, during the 3 months), based on the patient’s risk profile and personal preferences (e.g. quantified exercise plan, specific dietary measure, adherence to a new glucose-lowering therapy).

##### Behavioural contract

At the end of the consultation, the GP will fill out a one-page contract form that will summarise:

- The behaviour change goal
- The action plan
- The date of the next visit (after 3 months)

Participants will be asked to commit to the action plan by signing the contract form. A copy of the contract will be given to them, and another copy will be kept by the GP. Finally, participants will be invited to return to the practice 3 months later.

##### Feedback

At the follow-up visit, participants’ effective heart age will be updated. If behaviour change does not meet expectations, participants and GPs will discuss whether the behaviour change goal is appropriate and if the action plan needs to be applied more rigorously, modified, or abandoned (in which case alternative strategies will be considered). Another cycle will then start from that point onwards and up to the next follow-up visit.

Overall, the intervention will be implemented during three routine visits including the baseline visit — i.e. over 6 months — and be delivered besides standard diabetes care (see below).

#### Control group

Participants in the control group will receive standard diabetes care. In the absence of a concrete description in the Japanese Clinical Practice Guidelines [8], ‘standard diabetes care’ during visits will be defined as follows:

- Data gathering and review of the following items:
  - ◦ Symptoms (energy levels, polydipsia/polyuria, recurrent infections, vision, sensory disturbance/ weakness, sexual functioning, chest pain, shortness of breath)
  - ◦ Monitoring (medication adherence, immunisation uptake)
  - ◦ Social history (smoking, alcohol, diet, exercise, depression and anxiety, driving, occupation)
  - ◦ Red flags for diabetic ketoacidosis (vomiting, confusion, difficulty in breathing), hyperosmotic nonketotic coma (extreme thirst, polyuria, drowsiness, nausea, high/low blood sugars)
- Examination (body mass index, blood pressure, peripheral pulses, and foot care for infections, ulceration and footwear)
- Investigation (urine for urinalysis and microalbuminuria; blood for glucose, HbA1c, lipids, urea and creatinine)
- Request to make another appointment 3 months later

GPs in both groups will be asked to strictly adhere to these guidelines to make sure that ‘standard diabetes care’ is provided as consistently as possible across practices.

For comparison purposes regarding a certain number of secondary outcomes (see below), participants in the control group will also be communicated their personalised CV risks. However, this will take the form of a more conventional probabilistic risk information. They will receive a personalised CV risk score expressed as the 5-year risk probability of experiencing fatal or nonfatal coronary heart disease (CHD, i.e. angina pectoris or myocardial infarction) calculated by the JJ risk engine [51], and discuss appropriate risk reduction strategies with their GP. There will be no special criteria for discontinuing or modifying allocated interventions.

### Blinding

Study participants, GPs, and researchers in charge of data analysis will be blinded to treatment allocation. The study will be introduced as a comparison of the effects of two alternative types of intervention to ensure that GPs and participants in the control group remain blinded. Researchers in charge of GP trainings and data collection are the only party that cannot be masked to treatment allocation. The research team involves researchers from different institutions and countries, which allows one sub-team to conduct randomisation, another to liaise with GP practices and perform data collection, and a third to conduct data analysis.

### Outcomes

#### Primary outcome

The primary outcome will be the change in HbA1c levels at 6 months in the FEEDBACK intervention group compared with the control group. HbA1c levels will be derived from the samples collected by venepuncture at baseline, 3 months, and 6 months, obtained by the NGSP (National Glycohaemoglobin Standardisation Programme) certified method.

#### Secondary outcomes

##### Health-related outcomes

- The probability to achieve the recommended glycaemic target (HbA1c <7.0% [53mmol/mol]) at 6 months in each group will be compared.
- The change in participants’ CV risk score (5-year CHD probability) will be evaluated at 3 and 6 months. Personalised CV risk scores will be calculated by the JJ risk engine based on participants’ personal characteristics and risk factors, including age, sex, body mass index, duration of T2DM, smoking status, HbA1c level (%), systolic blood pressure (mm Hg), HDL cholesterol (mg/dL), total cholesterol (mg/dL), and urine albumin creatinine ratio (mg/gCre). Systolic blood pressure will be measured at the GP practice by the standard method recommended by the Japanese Society of Hypertension [52].
- The change in HbA1c levels will also be evaluated at 3 months.

##### Behavioural outcomes

- Changes in participants’ behavioural management will be measured using the Japanese-language version of the validated Summary of Diabetes Self-Care Activities Measure (SDSCA) instrument [53, 54] at each visit. The SDSCA contains 6 scales evaluating six dimensions of behavioural management: (1) dietary behaviour, (2) physical exercise, (3) SMBG, (4) medication adherence, (5) foot care, and (6) smoking. Higher scores reflect a greater number of days per week during which self-care activities are carried out (range 0–7). Only the scales numbered (1), (2), (4), and (6) will be used in this study.
- Changes in prescribed medications in terms of drug and/or dosage (increased, unchanged, or decreased) will also be recorded at each visit.

##### Psychosocial outcomes

- Doctor-patient communication will be evaluated using the dedicated 8-item subscale of a validated Japanese-language version of the General Practice Assessment Questionnaire (GPAQ) [55, 56] and a translated version of the 4-item ‘Ask, Understand, Remember Assessment’ (AURA) instrument [57, 58], after each visit. The GPAQ items focus on GP’s interpersonal and communication skills (patients will be asked to rate them on a scale of 1 to 6), while the AURA instrument assesses patient communication self-efficacy on a 1 to 4 scale.
- Participatory decision-making will be measured using a 7-item subscale of the validated Japanese-language version of the Patient Assessment of Chronic Illness Care (PACIC) scale, at baseline and at 6 months [59]. Participants will be asked how often over the past 6 months their GP performed any of six behaviours associated with participatory decision-making. The five response categories range from 0 (‘almost never’) to 5 (‘almost always’).
- Psychosocial self-efficacy will be measured at baseline, 3 and 6 months, using a validated Japanese-language version of the Appraisal of Diabetes Scale (ADS) [60, 61], a questionnaire developed to evaluate patients’ awareness of the psychological burden of T2DM and their ability to manage this burden. The ADS uses three subscales: four questions on the subjective impact of T2DM, two questions on the sense of self-control regarding T2DM, and one question on self-efficacy in diabetic control. Each item is rated on a 5-point Likert scale, with lower scores indicating better performance.
- Recall of the CV risk score (effective heart age or 5-year CHD probability) will be assessed as correct or incorrect based on open numerical responses (i.e. depending on whether or not the participant gives the exact right answer) immediately after the baseline visit, and at 3 months, immediately before the 2^nd^ visit.
- Emotional response to the CV risk score will be assessed by a 3-item, 7-point Likert scale, ranging from ‘not afraid/anxious/worried at all about the risk score’ to ‘very afraid/anxious/worried about the risk score’, immediately after the baseline visit [62].
- Perceived credibility of the CV risk score will be assessed by a 4-item (‘I felt that the number received was ‘my number’; ‘I found the number to be written personally for me’; ‘I felt that the information was relevant to me’; ‘I felt that the information was designed specifically for me’), 7-point Likert scale (from ‘completely disagree’ to ‘completely agree’) adapted from previous similar research, immediately after the baseline visit [63].
- Intentions to change behaviour according to the Theory of Planned Behaviour [64] will be measured using a translated version of the dedicated items of the Determinants of Lifestyle Behaviour Questionnaire (DLBQ) [65], before and after the baseline visit. Each item is associated with a different self-care activity (eating healthier, increasing physical activity, and quitting smoking) and is rated on a 5-point Likert scale, ranging from ‘strongly agree’ to ‘strongly disagree’.

Table 3 provides a summary of study outcome measures and assessment time points.

**Table 3:** Summary of outcome measures and assessment time points

**Outcome**	**Measurement method**	**Baseline visit**		**Follow-up (3 months)**		**Follow-up (6 months)**	
		Before visit	After visit	Before visit	After visit	Before visit	After visit
**Primary outcome**							
HbA1c level	Practice records	X				X	
**Secondary outcomes**							
*** Health-related outcomes***							
Probability to achieve the recommended glycaemic target	Practice records					X	
CV risk score	Practice records JJ risk engine	X		X		X	
HbA1c level	Practice records	X		X			
*** Behavioural outcomes***							
Behavioural management	SDSCA [53, 54]	X		X		X	
Prescribed medications	Practice record	X	X		X		X
**Psychosocial outcomes**							
Doctor-patient communication							
GP communication	GPAQ [55, 56]		X		X		X
Patient communication self-efficacy	AURA [57, 58]		X		X		X
Participatory decision-making	PACIC [59]	X					X
Psychosocial self-efficacy	ADS [60, 61]	X			X		X
Recall of CV risk score	Open-ended question		X	X			
Emotional response to CV risk score	Dedicated Likert scale [62]		X				
Perceived credibility of CV risk score	Dedicated Likert scale [63]		X				
Intentions to change behaviour	DLBQ [65]	X	X				

### Data collection

Eligible patients who agree to participate in the study following the face-to-face screening appointment will be notified of the start of the trial by the practice staff (i.e. once recruitment of all study participants is completed and all clusters are randomly allocated to intervention or control group). They will be informed that their next routine diabetes visit will be the baseline visit of the study.

On the day of the baseline visit, before seeing the GP, demographic information (age, sex, marital status, education level, employment status), diabetes-related characteristics (duration of T2DM, history of complications, current medications) and baseline measures will be collected using standardised forms or extracted from practice records by the practice staff. Baseline measures collected before the consultation include objective numeracy level assessed using the Japanese-language version of the Lipkus scale [66, 67] and relevant outcome measures described in Table 3. Right after the consultation, additional outcome measures will be collected (see Table 3).

The same procedure will apply at follow-up visits. The member of the practice staff in charge of data collection will make sure that the questionnaires are properly filled out and that no information is missing. Questionnaires will be linked to individual participants using practice and participant ID numbers that will preserve anonymity.

### Data analysis

#### Descriptive statistics

Participants will be described in terms of their baseline characteristics. Practice size (including both the total number of patients and the number of study participants) will also be reported. Continuous variables will be denoted using mean and standard deviation (normally-distributed variables) or median and interquartile range (non-normally distributed variables); while categorical variables will be denoted using count and percentage. Between-group differences in terms of participants’ characteristics and baseline measures will be tested using Student t tests and chi-square tests. In case significant between-group differences (*p*≤0.10) are observed, the relevant variables will be considered as potential confounders in further analysis.

#### Primary analyses for primary and secondary outcomes

The planned primary analyses will be carried out at the individual level, according to the intention-to-treat (ITT) principle. Between-group comparisons will be analysed using multilevel analysis to account for the dependency of observations. Between-group differences in the mean changes in primary and secondary outcomes will be estimated using linear mixed-effects models for continuous outcomes and logistic mixed-effects models for binary outcomes. Random effects will account for within-practice clustering and within-patient correlation. Fixed effects will be treatment group indicator (with the control group as the reference category), practice size (stratification variable) [68] and, where relevant, time indicators, their interaction effects, and baseline values of variables identified as potential confounders. In case normality assumptions are violated, outcome variables will be log-transformed and, if necessary, non-parametric tests will be used. Data analyses will be performed using Stata/MP 17 (StataCorp).

#### Sensitivity analyses

Sensitivity analyses will be carried out to evaluate the robustness of the estimated intervention effect on the primary outcome based on differing missing data assumptions. While our primary analytic approach can provide unbiased estimates under the assumption that data are missing at random, we will examine the sensitivity of statistical inferences to data being missing not at random, i.e. to cases where missingness depends on the unobserved values even after controlling for predictive variables. We will do so using a pattern-mixture model (PMM) approach, which assumes that participants who drop out have a mean outcome that deviates from that of participants who do not drop out by a given offset, and then explores the effect on the findings of various clinically realistic values of this offset (best case and worst-case scenarios) in the two study groups [69, 70].

#### Subgroup analyses

In addition, the ITT model for the primary outcome will be fitted for the following subgroups of interest: (1) including only patients with HbA1c > 8.0% at baseline to examine the effects of the FEEDBACK intervention on poorly controlled patients; (2) by duration of diabetes; (3) by age and sex; (4) by objective numeracy level; (5) by level of intention to change behaviour at baseline.

### Sample size

Assuming individual randomisation, recruitment of at least 76 participants per arm would be required to achieve 80% power with type I error α=0.05. These calculations have assumed the detection of a clinically meaningful difference of 0.5% (5.5 mmol/mol) in change in HbA1c between the two arms [71] and standard deviation of 1.1% (12 mmol/mol) based on a previous study with comparable population [72]. However, this sample size needs to be inflated to account for clustering effects and potential dropouts. Therefore, the final sample size will be adjusted according to the final number of clusters, the cluster average size, the estimated intra-class correlation coefficient (ICC), and the expected drop-out rate.

A systematic search of ICCs used in the design of cluster RCTs in T2DM with HbA1c as primary (continuous) outcome found that the median value used in published trials was 0.047 [73]. However, recent studies investigating the actual ICCs from large primary care datasets reported values comprised between 0.02 and 0.032 for HbA1c [74], suggesting that ICCs used in sample size calculation for cluster RCTs tend to be over conservative. Assuming an ICC of 0.035 and accounting for potential variations in cluster size, the effective sample size required to detect a 0.5% difference in HbA1c and to achieve 80% power with type I error α=0.05 is 119 participants per group. These calculations have assumed the mean cluster size to be 15 with standard deviation of 5, yielding a coefficient of variation of 0.33 and a design effect of 1.55 [75]. Moreover, assuming 10% loss to follow-up, 264 participants in total will be needed.

Based on these calculations, we aim to enrol 20 practices in the study. After 10 practices are enrolled (the inclusion of a minimum of 10 clusters is recommended, otherwise it is difficult to maintain both an appropriate type I error and sufficient power, even when small-sample correction methods are applied [76]), each practice will proceed with the recruitment of eligible patients. Two months after the start of the recruitment process, we will carry out a first assessment of each cluster size, calculate the coefficient of variation, and determine whether the total number of enrolled participants is sufficient according to the updated sample size calculation. Should this number be too small in view of the required sample size, we will recruit additional clusters until an effective sample size is reached, as long as our budget constraints permit it. In the unlikely event that additional practices cannot be recruited, or that the coefficient of variation in cluster size turns out to be much higher than expected (making the effective sample size too big given our budget constraint), we will consider opting for a cluster design with providers, rather than practices, as the unit of randomisation.

### Intervention fidelity

A standardised training protocol will be developed to minimise variability and maintain intervention protocol fidelity across all practices. GPs participating in the study will receive training consisting of a video featuring a mock consultation, delivered by a member of the research team to ensure that each step of the FEEDBACK intervention (or its control counterpart) is understood and appropriately followed. The members of the practice staff in charge of data collection will also receive instructions on how to properly fill out the standardised forms. Before the start of the trial, the intervention will be piloted in each practice allocated to the intervention group. We will assess the acceptability of the intervention and the feasibility of the data collection process. Should they be required, adjustments to the study procedures will be made.

### Confidentiality

In order to ensure data anonymity, practice and participant ID numbers will be used to combine datasets (baseline and follow-up measures) and to identify participants who withdraw from the study. The research team will not have access to the files enabling to match individual participants to their ID numbers. Participant data (i.e. consent forms, questionnaires, data extracted from practice records) will be stored in a locked cabinet in a researcher’s office. All data will be destroyed by the research team within 5 years after publication.

### Trial management

The study has two oversight committees:

- Trial Steering Committee (TSC)

This group is composed of the Principal Investigator, Co-Principal Investigator, study statistician, sponsor’s representative, and two members who are completely independent of the study. The TSC plays a critical role in providing strategic oversight and executive decision-making power in relation to the conduct of the study.

- Trial Management Team (TMT)

The day-to-day running of the trial will be overseen by a group chaired by the Principal Investigator and comprising of the data collection team and the partners of participating GP practices. This group will convene every two weeks to review the trial's progress, discuss any issues that have arisen, and ensure the smooth operation of the trial.

### Data monitoring

Due to the low-risk nature of the intervention, the short duration of the trial, and the fact that interim analyses may not provide necessarily informative results given the nature of the study, there will be no Data Monitoring Committee. The research team will comply with data collection and management procedures approved by the research ethics committee of Kashima Hospital, Fukushima, Japan, and abide by the rules of medical confidentiality.

### Harms

There is no anticipated harm resulting from participation in the trial, and participants will not receive compensation for their involvement. Nevertheless, some participants may express higher emotional response to CV risk communication than others. As emotional response to CV risk score is one of the secondary outcome measures, we will be able identify such participants and inform GPs to make sure that no unnecessary anxiety is triggered as a result of study participation. Should the study provide evidence of the effectiveness of the FEEDBACK intervention, we will provide access to it to study participants in the control group, to ensure they have access to the same benefits as those in the intervention group.

### Premature termination of the study

Given the short duration of the trial and the minimal risks associated with participation, no particular stopping rules have been set. In particular, we did not consider any stopping rule based on interim analyses. Although we will evaluate changes in HbA1c at the 3-month mark, we do not believe the lack of detectable effect at this stage to be an appropriate criterion for stopping the trial for futility reasons due to the fact that behavioural changes may take a longer period of time to be reflected in HbA1c levels. Any decision to stop the study will be made by the TSC based on recruitment rate or adverse events.

### Auditing

There will be no independent audit of the trial. The research team and participating GPs will manage security and quality according to their standard operating procedure.

## Discussion

This article describes the design of a cluster RCT that will evaluate the effects of FEEDBACK, a personalised, multicomponent intervention aimed at engaging adults with T2DM in behavioural management more effectively. The intervention has been developed to be easily embedded into routine primary care consultation delivered in an outpatient clinic setting. Following Peyrot and Rubin’s 5C framework [19], practical considerations have been a central aspect of its development process. Our objective was to minimise the time and resources needed to deliver the intervention so that it could be easily adopted by GPs, integrated into regular consultation, and also scalable in a wide range of clinical settings. Primary care is the key contact point with the healthcare system for people with T2DM in many countries around the world. In Japan, where the implementation of primary care policy has just begun, it is necessary to introduce evidence-based, person-centred diabetic care into the practice of GPs nationwide. If the proposed behaviour change programme leads to improved outcomes and delivery in primary care, this would provide further justification for stronger primary care services and investment in primary care in Japan.

The intervention uses several BCTs that have shown promise in promoting behavioural management of T2DM, such as ‘goal setting’, ‘problem-solving’, and ‘action planning’. It also operates two BCTs that have, to our knowledge, not yet been tested for this purpose: communicating CV risks using the risk metric ‘effective heart age’ (thereby leveraging the ‘salient consequences’ of suboptimal diabetes control) and ‘contracting for behaviour change’. The development of ‘effective age’ tools to communicate health risks more intuitively has recently attracted increasing attention [77], including in the T2DM area [49]. A recent feasibility study conducted with people with T2DM receiving primary care management in the UK suggests that communicating heart age has promising effects on risk recall in patients with poor glycaemic control [78]. However, evidence of the effects of such tools on actual behaviour change is still scarce, with existing studies reporting mixed results. For example, Lopez-Gonzalez and colleagues found greater reductions in lifestyle and clinical risk factors for CVD after 12 months when people were shown an interactive heart age tool by their clinician compared to usual care or communicating 10-year absolute risk in verbal format [79]. Bonner and colleagues, on the other hand, did not find any evidence that heart age motivates lifestyle change more than 5-year absolute risk in individuals with low CVD risk [63]. There is a need for methodologically robust studies to inform which patient populations are more likely to benefit from age-based risk formats and whether such tools should be embedded more widely into clinical practice [77].

Importantly, the effectiveness of BCTs in promoting behavioural management of T2DM may vary across populations. Most studies having been conducted in Western countries so far (e.g. 9 out of the 13 RCTs included in a recent systematic review [27]), there is a need to generate more evidence from non-Western populations and settings to identify which techniques work best in different contexts. This study also aims to contribute to filling this gap, at a time where T2DM has become highly prevalent in most regions of the world. In Japan, more than 10 million adults are currently suspected to be affected by the condition (16.3% of males and 9.3% of females) [80] and, despite population decline in the country, its prevalence has been continuously growing since 2005 and is unlikely to revert in the near future [81].

Changes in HbA1c levels between the intervention and control groups will be compared at the individual level, following three visits during which the intervention (or its control counterpart) will be delivered. If proven effective in improving glycaemic control, the FEEDBACK intervention will be relevant to policymakers and clinicians concerned with reducing the burden of T2DM while also minimising programme costs by building on available resources and existing infrastructures. A major strength of this study lies in a careful study design, with recruitment of individual participants being carried out before randomisation of practices to reduce the risk of selection bias. Although this is recommended by international guidelines [38], recruitment constraints in cluster trials often make it challenging and many published studies have not followed the guidelines on this very point [39]. Limitations of the study include the impossibility to blind GPs to treatment allocation due to the nature of the intervention, and the use of self-reported questionnaires to measure behavioural management. Although the SDSCA instrument has acquired the status of ‘gold standard’, feasibility studies have shown that participants may encounter difficulties in answering some of its questions (e.g. when asked to quantify their daily intake of fruits and vegetables) [78].

## Trial status

Protocol date: 18/01/2023. Protocol version: 1.0. Recruitment of participants started in December 2022 and is expected to be completed in April 2023. Data collection is scheduled to be completed by March 2024, after which the final data analysis will be performed and the findings presented in a separate publication.

## Supplementary Information

**Additional file 1.**

## Data Availability

The research team will have exclusive rights to the de-identified data for 24 months after the trial is completed. After that, the datasets, statistical codes, and full protocol of the study will be available to interested parties upon reasonable request to the corresponding author.

## Abbreviations

BCT: Behaviour change technique
CHD: Coronary heart disease
CV: Cardiovascular
CVD: Cardiovascular disease
FEEDBACK: Fukushima study for Engaging people with type 2 Diabetes in Behaviour Associated Change
GP: General practitioner/family physician
HbA1c: Haemoglobin A1c
ICC: Intra-class correlation coefficient
ITT: Intention-to-treat
JPCA: Japan Primary Care Association
NGSP: National Glycohaemoglobin Standardisation Programme
PMM: Pattern-mixture model
RCT: Randomised controlled trial
SMBG: Self-monitoring of blood glucose
T2DM: Type 2 diabetes mellitus
WONCA: World Organization of National Colleges, Academies and Academic Associations of General Practitioners/Family Physicians (World Organization of Family Doctors)
